# Acute neuropathological consequences of short-term mechanical ventilation in wild-type and Alzheimer’s disease mice

**DOI:** 10.1186/s13054-019-2356-2

**Published:** 2019-02-22

**Authors:** Shouri Lahiri, Giovanna C. Regis, Yosef Koronyo, Dieu-Trang Fuchs, Julia Sheyn, Elizabeth H. Kim, Mitra Mastali, Jennifer E. Van Eyk, Padmesh S. Rajput, Patrick D. Lyden, Keith L. Black, E. Wesley Ely, Heather D. Jones, Maya Koronyo-Hamaoui

**Affiliations:** 1 Department of Neurology, Cedars-Sinai Medical Center, 127 S. San Vicente Blvd., AHSP Building, Suite A6600, A8103, Los Angeles, CA 90048 USA; 2 Department of Neurosurgery, Cedars-Sinai Medical Center, 127 S. San Vicente Blvd., AHSP Building, Suite A6600, A8103, Los Angeles, CA 90048 USA; 3 Department of Biomedical Sciences, Cedars-Sinai Medical Center, 127 S. San Vicente Blvd., AHSP Building, Suite A6600, A8103, Los Angeles, CA 90048 USA; 4 Biostatistics and Informatics Core, Cancer Institute, Cedars-Sinai Medical Center, Los Angeles, USA; 5 Department of Neurology, Cedars-Sinai Medical Center, Los Angeles, USA; 6 Department of Pulmonary and Critical Care Medicine, Veteran’s Affairs Tennessee Valley Geriatric Research Education and Clinical Center, Vanderbilt University School of Medicine, Nashville, USA; 7 Biomedical Imaging Research Institute, Cedars-Sinai Medical Center, Los Angeles, USA

**Keywords:** Mechanical ventilation, Cognitive impairment, Alzheimer’s disease, Critical illness

## Abstract

**Background:**

Mechanical ventilation is strongly associated with cognitive decline after critical illness. This finding is particularly evident among older individuals who have pre-existing cognitive impairment, most commonly characterized by varying degrees of cerebral amyloid-β accumulation, neuroinflammation, and blood-brain barrier dysfunction. We sought to test the hypothesis that short-term mechanical ventilation contributes to the neuropathology of cognitive impairment by (i) increasing cerebral amyloid-β accumulation in mice with pre-existing Alzheimer’s disease pathology, (ii) increasing neurologic and systemic inflammation in wild-type mice and mice with pre-existing Alzheimer’s disease pathology, and (iii) increasing hippocampal blood-brain barrier permeability in wild-type mice and mice with pre-existing Alzheimer’s disease pathology.

**Methods:**

We subjected double transgenic Alzheimer’s disease (APP/PSEN1) and wild-type mice to mechanical ventilation for 4 h and compared to non-mechanically ventilated Alzheimer’s disease model and wild-type mice. Cerebral soluble/insoluble amyloid-β_1–40_/amyloid-β_1–42_ and neurological and systemic markers of inflammation were quantified. Hippocampal blood-brain barrier permeability was quantified using a novel methodology that enabled assessment of small and large molecule permeability across the blood-brain barrier.

**Results:**

Mechanical ventilation resulted in (i) a significant increase in cerebral soluble amyloid-β_1–40_ (*p* = 0.007) and (ii) significant increases in neuroinflammatory cytokines in both wild-type and Alzheimer’s disease mice which, in most cases, were not reflected in the plasma. There were (i) direct correlations between polymorphonuclear cells in the bronchoalveolar fluid and cerebral soluble amyloid-β_1–40_ (*p* = 0.0033), and several Alzheimer’s disease-relevant neuroinflammatory biomarkers including cerebral TNF-α and IL-6; (iii) significant decreases in blood-brain barrier permeability in mechanically ventilated Alzheimer’s disease mice and a trend towards increased blood-brain barrier permeability in mechanically ventilated wild-type mice.

**Conclusions:**

These results provide the first evidence that short-term mechanical ventilation independently promotes the neuropathology of Alzheimer’s disease in subjects with and without pre-existing cerebral Alzheimer’s disease pathology. Future studies are needed to further clarify the specific mechanisms by which this occurs and to develop neuroprotective mechanical ventilation strategies that mitigate the risk of cognitive decline after critical illness.

**Electronic supplementary material:**

The online version of this article (10.1186/s13054-019-2356-2) contains supplementary material, which is available to authorized users.

## Background

Mechanical ventilation is considered a life-saving critical care intervention that is implemented in nearly 800,000 patients annually in the USA [1]. It is now recognized that up to 40% of these patients acquire new and significant cognitive impairment that often resembles the phenotype of Alzheimer’s disease (AD), a neurodegenerative condition characterized by cerebral amyloid β (Aβ) peptide accumulation, neuroinflammation, and blood-brain barrier dysfunction [2, 3]. It is also known that the risk for cognitive decline after critical illness is particularly high in individuals with pre-existing cognitive impairment, of which AD is the most common type [4, 5].

It has been hypothesized that mechanical ventilation may contribute to long-term cognitive impairment by promoting cerebral accumulation of the Aβ peptide, systemic and neurologic inflammation, and blood-brain barrier dysfunction although the exact mechanisms remain unknown [6–9]. To our knowledge, no prior studies have examined the effects of mechanical ventilation in models of AD, which is relevant since most patients who undergo mechanical ventilation are elderly, and thus at higher risk for carrying increased cerebral Aβ pathology [1, 8, 10–12].

Accordingly, in this study, we sought to test the following hypotheses: (i) short-term injurious mechanical ventilation increases cerebral Aβ accumulation in the double transgenic APP_SWE_/PSEN1_∆E9_ (ADtg) mouse models of AD, (ii) short-term mechanical ventilation increases systemic and neurologic inflammation in wild-type and ADtg mice, and (iii) short-term mechanical ventilation increases blood-brain barrier permeability of small or large-size molecules in wild-type and ADtg mice.

## Methods

### Mice

Double transgenic mouse models of Alzheimer’s disease (ADtg) from the B6Cg-Tg (APP_SWE_, PSEN1_∆E9_) 85Dbo/J strains, and their sex- and age-matched non-transgenic wild-type littermates, were purchased from Jackson Laboratories (MMRC stock #34832-JAX|APP/PS1), then bred and maintained at Cedars-Sinai Medical Center in common environmental conditions. ADtg mouse model harbors both the chimeric mouse/human APP (APP_SWE_) and the mutant human presenilin 1 (PSEN1_∆E9_) genes [13, 14]. A total of 41 mice (4 to 6.5 months old and 24.1 to 29.7 g in weight) were used: 21 ADtg mice and 20 wild-type mice. Of the ADtg mice, 12 were mechanically ventilated and 9 were not mechanically ventilated (spontaneous breathing) and of the 20 wild-type mice, 8 were mechanically ventilated and 12 were not mechanically ventilated. All experiments were conducted in accordance with Cedars-Sinai Medical Center Institutional Animal Care and Use Committee (IACUC) guidelines under an approved protocol and complied with the current US law.

### Genotyping

Genomic DNA was extracted from a tail or ear tip using a DNA extraction kit (Qiagen) following the manufacturer’s protocol. Mice used in this study were genotyped for the presence of the transgenes by PCR, as previously described [15].

### Intubation and mechanical ventilation

Mice to be mechanically ventilated were anesthetized with intraperitoneal injection of a mix of ketamine (Vedco Inc., Saint Joseph, MO, USA) and dexmedetomidine (Pfizer, Irvine, CA, USA) (75 mg/kg and 0.5 mg/kg, respectively). Mice were orotracheally intubated and ventilated for 4 h using an Inspira volume-controlled small animal ventilator (Harvard Apparatus, Holliston, MA, USA) with a tidal volume of 15 ml/kg and a respiratory rate of 70 breaths per minute, with zero PEEP. Subcutaneous PBS (500 μl) was administered at the onset of mechanical ventilation and every 3 h of mechanical ventilation thereafter. Fifty milligrams per kilogram of ketamine was administered as subcutaneous injections as needed, usually every 2–3 h. Mice were kept warm on a 38 °C heating pad (Hallowell EMC, Pittsfield, MA, USA). Anesthesia was reversed with atipamezole (1 mg/kg in 100 μL of sterile water), and the animals were allowed to recover in their cages on a heating pad for 6 h. Control mice remained in their cages until the end of mechanical ventilation, at which time they were euthanized together with the ventilated mice.

### Pulse oximetry

Arterial oxygen saturations were measured in mechanically ventilated mice using the MouseOX pulse oximeter (STARR Life Sciences, Oakmont, PA, USA).

### Tracer infusion to assess BBB permeability

Following mechanical ventilation, mice recovered for 6 h after which each was administered 50 μL of intravenous Texas Red-dextran 3 kD (0.25%) and FITC-dextran 2000 kD (0.25%) via tail vein and subsequently euthanized by perfusion 30 min later. Non-mechanically ventilated control animals also underwent tracer infusion and euthanasia by perfusion 30 min later.

### Isolation of brain tissue and blood collection

At experimental completion, mice were deeply anesthetized and perfused with ice-cold saline supplemented with 0.5 mM EDTA. Blood was collected directly from the vena cava prior to cardiac perfusion. The left hindbrains were collected and stored at − 80 °C for further protein analysis, and the right hemispheres were fixed in 2.5% paraformaldehyde (Sigma-Aldrich) and cryo-protected in 30% sucrose for further histological analysis. For the latter, 30-μm-thick coronal brain sections were prepared.

### Bronchoalveolar lavage

Immediately after sacrificing the animal by perfusion, the trachea was exposed and cannulated with a 22-G intravenous cannula. PBS (0.5 ml) with 2 mM EDTA was instilled and aspirated twice. Cells were separated from the supernatant and immediately analyzed for cell counts.

### Primary endpoint: cerebral Aβ quantification using enzyme-linked immunosorbent assay (ELISA)

Fresh frozen left hindbrains were thoroughly homogenized in PBS buffer with 0.5% Triton X-100 (Sigma) and 1% protease inhibitor cocktail set I (Calbiochem), then processed as previously described [16]. After determination of protein concentration using the Pierce BCA Protein Assay Kit (Thermo Scientific), concentrations of Aβ_1–42_ and Aβ_1–40_ in the soluble and insoluble brain protein fractions were analyzed with sandwich ELISA kit (Invitrogen), per manufacturer’s instructions. The optical density of each well was read at 450 nm using the same microplate reader (Spectra Max 384 plus, Molecular Devices).

### Immunohistochemistry (IHC)

Paraformaldehyde-fixed cryo-sections of the coronal brain were treated for 30 min in antigen-retrieval solution (Dako) prior to serum-free protein blocking (Dako Cytomation). Sections were then hybridized with various primary antibodies (overnight at 4 °C): mouse anti-human Aβ [residues 1–16, mAb clone 6E10 (1:100; Covance)], rat anti-GFAP pAb (1:1000; Sigma-Aldrich), and rabbit anti-CD31 pAb (1:50; Abcam). Hybridization with primary antibodies was followed by incubation with fluorophore-conjugated secondary antibodies (1 h at 37 °C; donkey anti-mouse, anti-rat, and anti-rabbit; 1:200; Jackson Immuno Research Laboratories) conjugated with Cy2, Cy3, and Cy5. Sections were mounted using ProLong Gold with DAPI (Molecular Probes, Life Technologies). Negative controls were processed using the same protocol with the omission of the primary antibody to assess non-specific labeling. A Carl Zeiss Axio Imager Z1 fluorescence microscope equipped with ApoTome (Carl Zeiss MicroImaging, Inc.) was used for microscopic analysis. AxioVision (release 4.6.3) software (Carl Zeiss) was used to process and analyze the images.

### Secondary measure: systemic and cerebral inflammatory biomarkers quantification using Meso Scale Discovery (MSD) multiplex inflammatory assay

MSD V-PLEX proinflammatory panel I and cytokine panel 1 (mouse) are highly sensitive multiplex ELISA which measures quantitatively 19 cytokines (interferon γ (IFN-γ), interleukin (IL)-1β, IL-2, IL-4, IL-6, IL-8, IL-10, IL-12p70, IL-13, tumor necrosis factor α (TNF-α), IL-9, MCP-1, IL-33, IL-27p28/IL-30, IL-15, IL-17A/F, MIP-1α, IP-10, and MIP-2) using electrochemiluminescence detection technology (Meso Scale Discovery [MSD], Rockville, MD, USA). Both plasma and brain tissue lysates were prepared according to MSD protocol and were measured in duplicate: 50 μl of twofold diluted samples and calibrators were pipetted in each well and incubated for 2 h at room temperature with shaking. Following washing the plates three times with wash buffer (1× PBS and 0.05% Tween20), 25ul of MSD detection antibody solution was added to each well and incubated for 2 h at room temperature with shaking. Plates were washed three times with wash buffer and then 150 μl of MSD Read Buffer was added to each well. The plates then were read and analyzed by the MSD instrument (MESO Quicklex S 120).

### Secondary measure: fluorescent tracer quantification to assess blood-brain barrier permeability

Blood-brain barrier permeability was assessed using confocal microscopy to assess extravasation of Texas Red-dextran (3 kD) and FITC-dextran (2000 kD) tracer injected 30 min prior perfusion (0.25% each; see above).

Five consecutive hippocampal sections were imaged per mouse using FLV10 FluoView microscope at exposure DAPI 0.0133, GFP 1.240, and Texas Red 15.49. Quantification was achieved in ImageJ (NIH) by selecting regions of interest in the hippocampus. For FITC channel images, the background was subtracted to 10 pixels, the contrast was set to 10–60, and the threshold at 15–255. For Texas Red images, the contrast was set at 12–50 first, and then background subtraction to 5 pixels followed by thresholding at 50–255. Data of area fraction (%) was summed for each animal and then averaged per treatment group.

### Statistical analysis

Data were analyzed using GraphPad Prism vr. 7.01 (GraphPad) Software. Two-way ANOVA was used to compare mean differences between two or more groups when potential interactions with independent variables, such as genotype or time, could affect the measured outcomes. Following two-way ANOVA, Holm-Sidak’s post hoc test was applied to assess pairwise comparisons between experimental groups while correcting for multiple comparisons. Unpaired two-tailed Student’s *t* tests were used in two-group comparisons for matched experimental groups. ADtg mice were age- and sex-matched (*n* = 6–9 mice/group) to enable comparisons of Aβ concentrations and blood-brain barrier permeability. The statistical association between two or more variables was determined by Pearson’s correlation coefficient (*r*) test (GraphPad Prism), with corrections for multiple analyses. Pearson’s *r* indicates direction and strength of the linear relationship between two variables. Results are shown as means ± standard errors of the mean (SEMs). Degrees of significance between groups are represented as follows: *p < 0.05, **p < 0.01, ***p < 0.001, and ****p < 0.0001. A *p* value of less than 0.05 was considered significant. Post hoc Bonferroni adjustment was performed to correct for multiple comparisons for the neuroinflammatory biomarker data and altered the threshold for significance to *P* < 0.0028. Only the laboratory staff member performing the experimental intervention was aware of the experimental group allocations. Data analysts were provided with coded identifiers and remained blinded to experimental group allocations until after completion of all analyses.

## Results

**Fig. 1:**
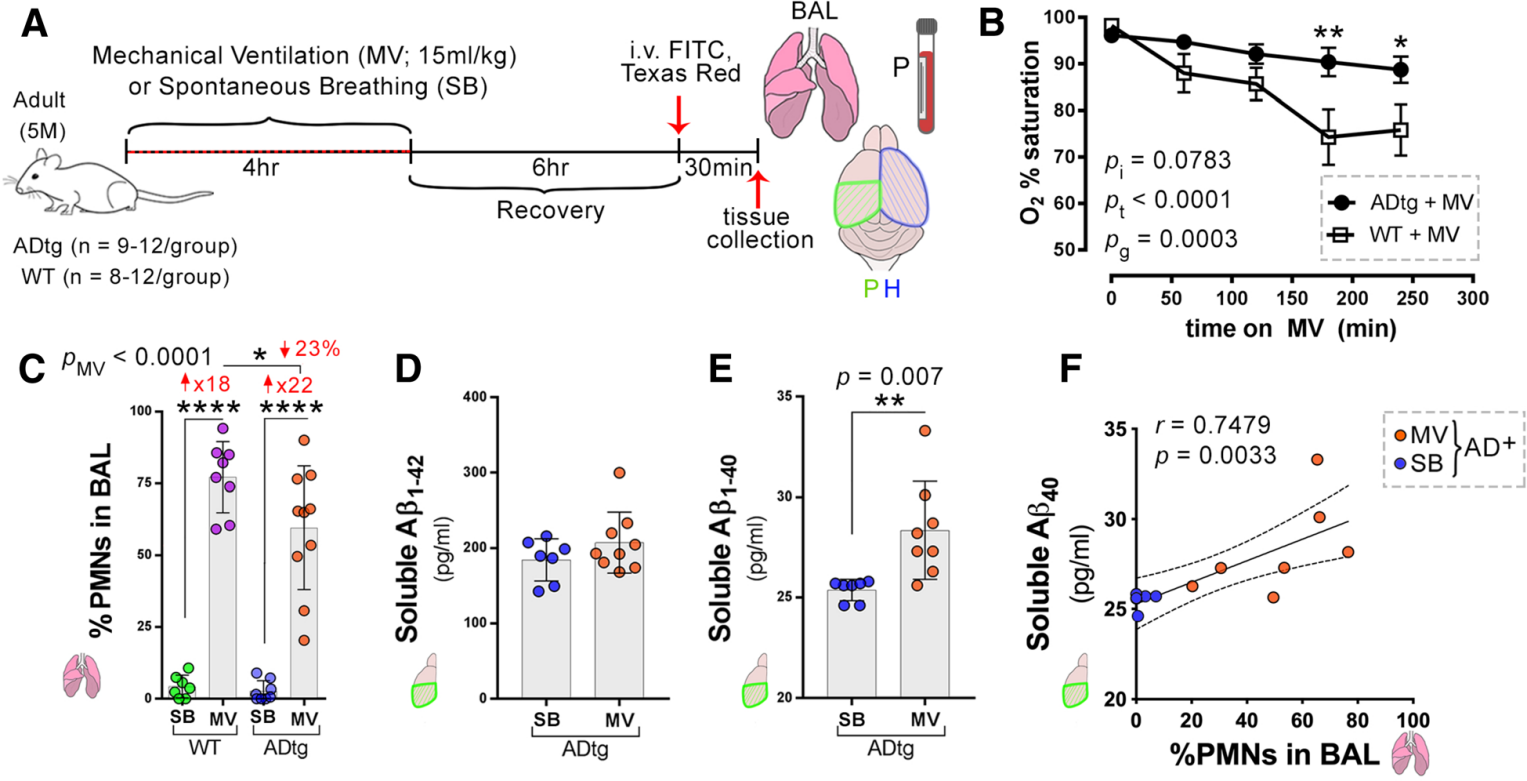
Mechanical ventilation induces pulmonary inflammation and increases cerebral soluble Aβ_1–40_ in ADtg mice. **a** Schematic illustration of experimental design and timeline: 5-month-old ADtg and wild-type (WT) mice underwent mechanical ventilation (MV) with a tidal volume of 15 ml/kg for 4 h while control groups of ADtg and WT mice were spontaneously breathing (SB) (*n* = 8–12 mice per group). Mice recovered for 6 h and 30 min prior to perfusion, they received intravenous injections of Texas Red-dextran (3 kD) and FITC-dextran (2000 kD) tracers (0.25% each). Bronchoalveolar lavage (BAL) fluid specimens were analyzed for cell count, plasma and left hindbrains were collected for protein analysis (P), and the right brain hemispheres were isolated for histology (H). **b** Mean arterial oxygen saturation for ADtg and WT mice are presented for each hour of MV, analyzed by two-way ANOVA. **c** Percent of polymorphonuclear cells (PMNs), or neutrophils, in the BAL was measured for each group, ADtg and WT in both MV and SB conditions (*n* = 8–12 mice/group). **d** Sandwich ELISA analysis of human soluble Aβ_1–42_ levels in the brains of age-matched ADtg mice (*n* = 8–9 mice/group). **e** ELISA analysis of cerebral soluble Aβ_1–40_ levels in age-matched ADtg mice (*n* = 8–9 mice/group). **f** Pearson’s *r* correlation analysis between cerebral soluble Aβ_1–40_ and % PMNs in BAL in age-matched ADtg mice in both conditions, MV (orange dots) and SB (yellow dots) with 95% confidence interval (CI) in dashed lines. Data from individual mice and group means with standard error of measurements are shown, as well as *p* values (*p*_i_ = *p* value for interaction; *p*_MV_ = *p* value for MV intervention effect; *p*_g_ = *p* value for genotype effect). Fold increases in MV compared to SB-control groups are shown in red. *p < 0.05, **p < 0.01, ****p < 0.0001, using two-way ANOVA with Holm-Sidak’s post hoc multiple comparisons correction, unpaired two-tailed Student *t* tests for two-group comparison, and Pearson’s correlation analysis

An overview of the experimental timeline is shown in Fig. 1a. ADtg mice (*n* = 12; mean age 4.9 ± 0.8 months and weight 26.2 ± 2.9 g) and wild-type mice (*n* = 8; mean age 5.3 ± 0.8 months and weight 26.3 ± 3.7 g) underwent mechanical ventilation with a tidal volume of 15 ml/kg for 4 h, while control groups of non-mechanically ventilated ADtg mice (*n* = 9; mean age 4.9 ± 1.8 months and weight 27.8 ± 3.2 g) and WT mice (*n* = 12; mean age 5.7 ± 1.1 months and weight 27.5 ± 1.2 g) were not mechanically ventilated. There were no significant differences in age or weight between any of the groups (*p* > 0.05). There was a decrease in mean oxygen saturation during the 4 h of mechanical ventilation in both ADtg and wild-type mice. In addition, the mean oxygen saturation was significantly lower in the wild-type mice compared to the ADtg mice (Fig. 1b). Polymorphonuclear (PMN) cell percent was measured in the bronchoalveolar fluid to assess the extent of pulmonary inflammation due to mechanical ventilation and was significantly increased in the mechanically ventilated wild-type and ADtg mice (Fig. 1c). We then quantified soluble Aβ_1–40_ and Aβ_1–42_ peptides, which are closely associated with the pathogenesis of AD [3]. There was a significant increase in cerebral concentrations of soluble Aβ_1–40_ in age- and sex-matched ADtg mice subjected to mechanical ventilation compared to those who did not undergo mechanical ventilation (Fig. 1e), and there was a direct correlation between soluble Aβ_1–40_ and percent PMN cells in the bronchoalveolar fluid (Fig. 1f). There was a non-statistically significant increase in soluble Aβ_1–42_ of ADtg mice subjected to mechanical ventilation compared to those who did not undergo mechanical ventilation (Fig. 1d).

**Fig. 2:**
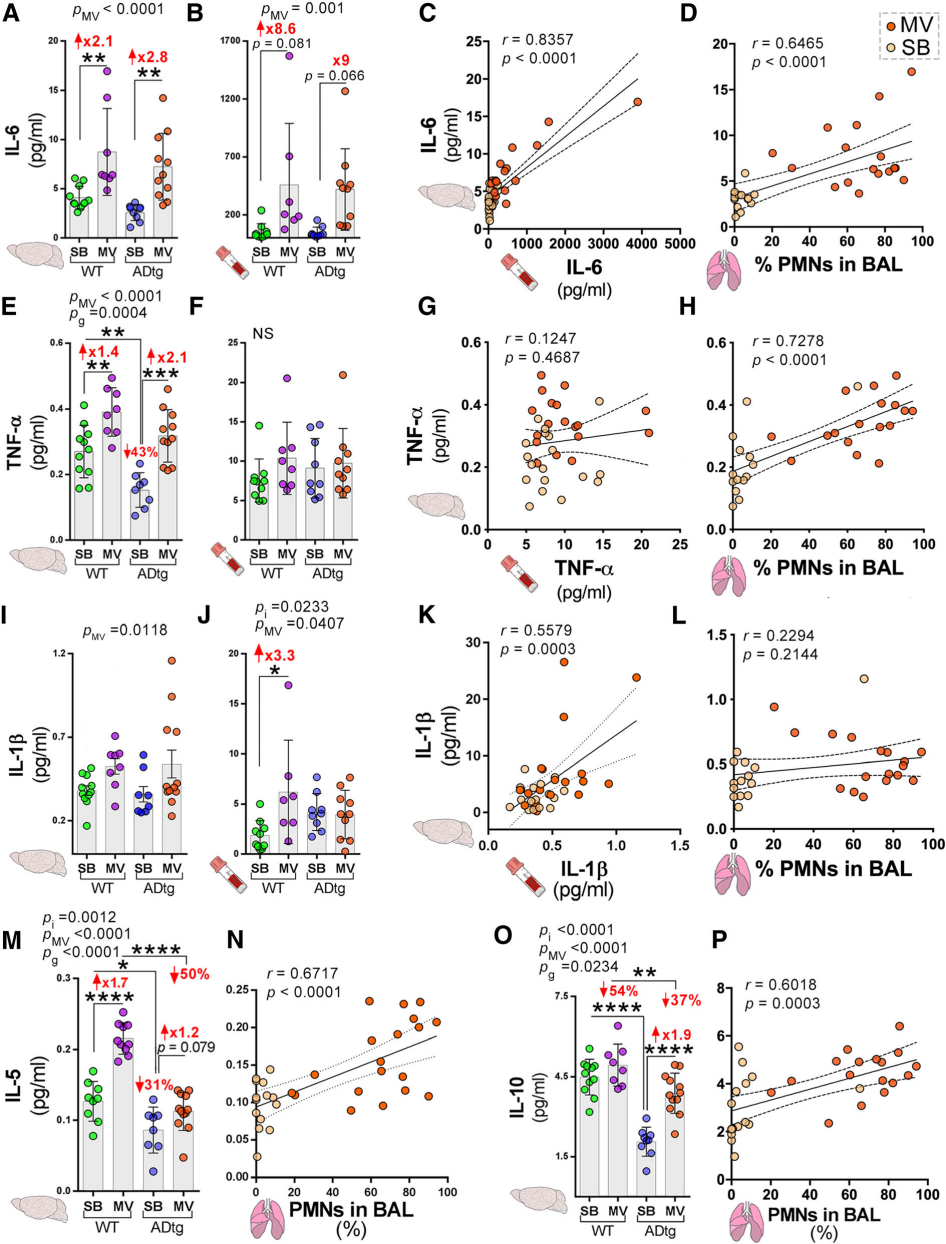
Mechanical ventilation affects key cognition-relevant cytokine responses in the brain and plasma of WT and ADtg mice. The Meso Scale Discovery (MSD) multiplex inflammatory assay was performed on plasma and brain specimens from all experimental groups (*n* = 8–12 mice/group). **a**, **b** IL-6 expression. **c** Pearson’s *r* correlation analysis between cerebral and plasma IL-6 levels and **d** cerebral IL-6 and % PMNs in BAL. **e**, **f** TNF-α expression. **g** Correlations between cerebral and plasma TNF-α levels and **h** cerebral TNF-α and % PMNs in BAL**. i**, **j** IL-1β expression. **k** Correlations between cerebral and plasma IL-1β levels and **l** cerebral IL-1β and % PMNs in BAL. **m** IL-5 expression in the brain. **n** Correlation between brain IL-5 and % PMNs in BAL. **o** IL-10 expression in the brain. **p** Correlation between brain IL-10 and % PMNs in BAL. Data from individual mice and group means with standard error of measurements are shown as well as *p* values (*p*_i_ = *p* value for interaction; *p*_MV_ = *p* value for MV intervention effect; *p*_g_ = *p* value for genotype effect); mice in the MV group are indicated by orange dots and mice in the SB group by yellow dots. Fold increase and percentage decrease between the groups are shown in red. *p < 0.05, **p < 0.01, ***p < 0.005, ****p < 0.0001, using two-way ANOVA with Holm-Sidak’s post hoc multiple comparisons correction or Pearson’s *r* correlation analysis with 95% CI

Figure 2 illustrates notable changes in cognition-relevant cytokines following mechanical ventilation in both mice strains (see Additional file 1: Figures S2–S7 for all other findings). In comparison to the non-mechanically ventilated mice, mechanically ventilated mice demonstrated significant increases in cerebral IL-6 and TNF-α in both mice strains (Fig. 2a, e). There was a significant increase in IL-5 in mechanically ventilated wild-type mice and a significant increase in IL-10 in mechanically ventilated ADtg mice (Fig. 2m, o). There was a trend towards increased plasma IL-6 following mechanical ventilation in both mice strains while plasma IL-1β increased significantly in the wild-type mice only (Fig. 2b, j). In contrast, there was no significant change in plasma TNF-α following mechanical ventilation and no significant correlation with cerebral TNF-α (Fig. 2f, g). There were direct and significant correlations between (i) PMN percent in the bronchoalveolar fluid and cerebral IL-6, TNF-α, IL-5, and IL-10 concentrations (Fig. 2d, h, n, p) and (ii) plasma and cerebral concentrations of IL-6 and IL-1β (Fig. 2c, k). There were significant interactions between genotype and mechanical ventilation for cerebral IL-5, IL-10, and plasma IL-1β (indicated by *p*_i_ in Fig. 2m, o, and j). Other significant interactions can be found in Additional file 2.

**Fig. 3:**
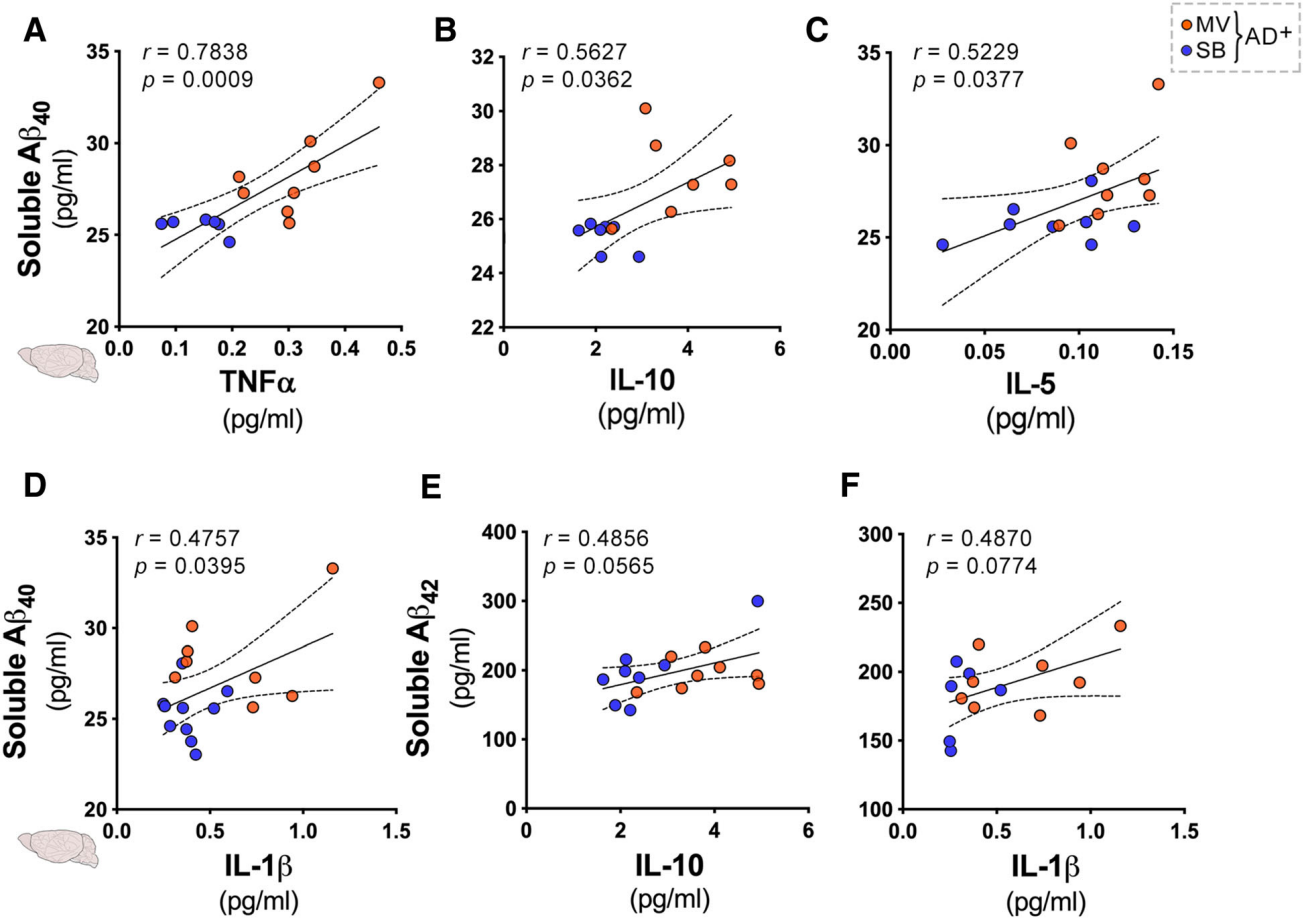
Associations between cerebral soluble Aβ and key cytokines in ADtg mice following mechanical ventilation. Pearson’s *r* correlations between human soluble Aβ_1–40_ and Aβ_1–42_ levels and cerebral cytokine levels measured by the Meso Scale Discovery (MSD) multiplex inflammatory assay in age-matched ADtg mice (*n* = 8–9 mice/group). **a**–**d** Correlations between cerebral soluble Aβ_1–40_ levels in ADtg groups (MV and SB) and the following cerebral cytokines: **a** TNF-α, **b** IL-10, **c** IL-5, and **d** IL-1β. **e**, **f** Correlations between cerebral soluble Aβ_1–42_ and the following cerebral cytokines: **e** IL-10 and **f** IL-1β. Data from individual mice in MV (orange dots) and SB (yellow dots) groups are shown. Both *r* and *p* values, as well as 95% CI, dashed lines are shown as measured by Pearson’s *r* correlations

Assessment of potential relationships between cerebral Aβ concentrations and cognition-relevant cytokines in ADtg mice revealed direct and significant correlations between (i) cerebral TNF-α and soluble Aβ_1–40_ (Fig. 3a), (ii) cerebral IL-10 and soluble Aβ_1–40_ (Fig. 3b), (iii) cerebral IL-5 and soluble Aβ_1–40_ (Fig. 3c), and (iv) cerebral IL-1β and soluble Aβ_1–40_ (Fig. 3d). There was no significant association between cerebral IL-10 and soluble Aβ_1–42_ and cerebral IL-1β and soluble Aβ_1–42_ (Fig. 3e, f).

**Fig. 4:**
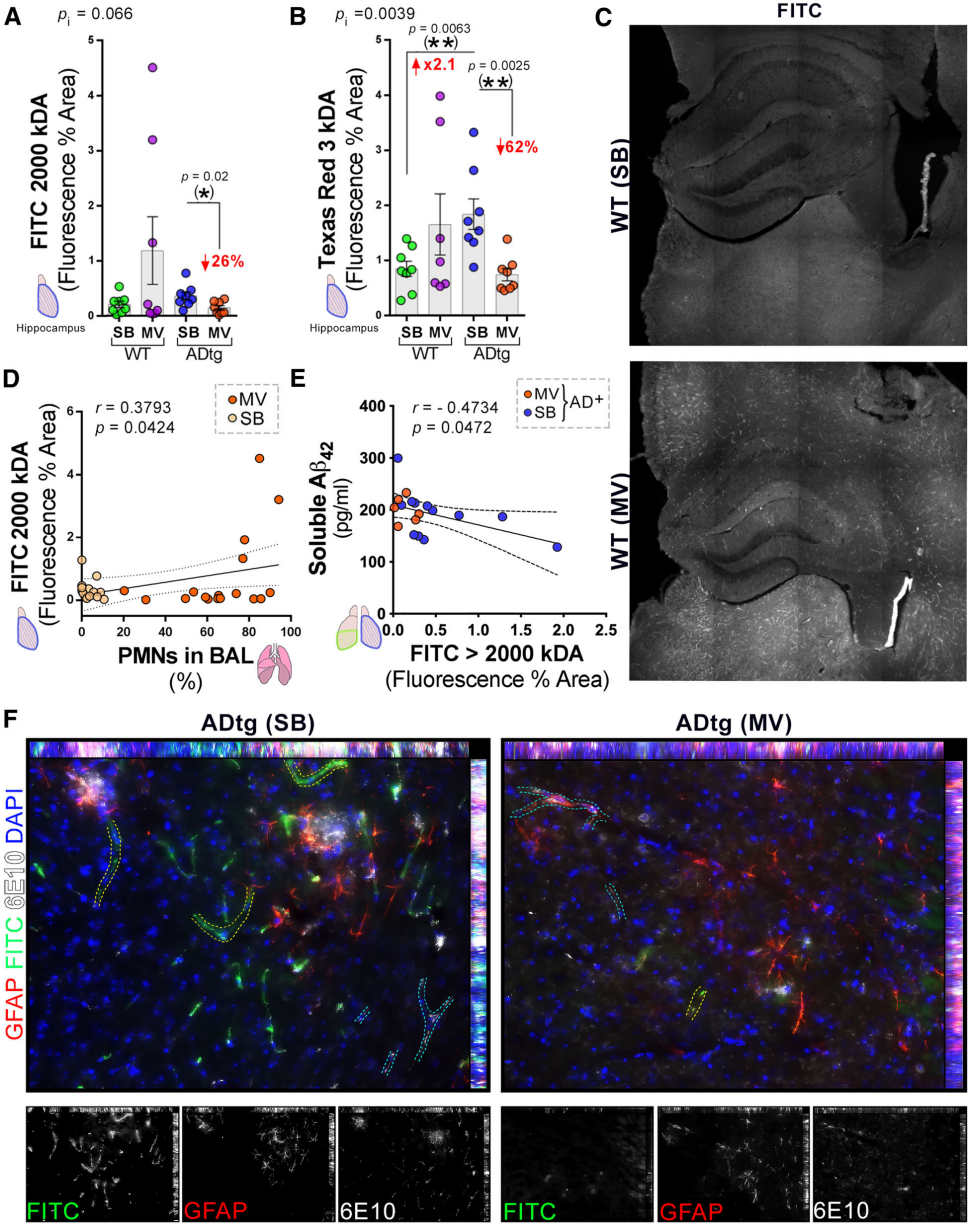
Blood-brain barrier permeability in the hippocampus following mechanical ventilation in WT and ADtg mice. Blood-brain barrier damage in the hippocampus was assessed by quantitative analysis of percent area of extravasation of either high molecular weight FITC-dextran (2000 kD) or low molecular weight Texas Red-dextran (3 kD) tracers. **a** FITC-dextran and **b** Texas Red-dextran. **c** Representative confocal images of FITC-dextran tracer in WT mice in the SB (top) and MV (bottom) conditions. **d**, **e** Pearson’s *r* correlation analysis between FITC-dextran hippocampal tracer leakage and **d** % PMNs in BAL of all animals (*n* = 8–12 mice/group) and **e** soluble cerebral Aβ_1–42_ in both groups of ADtg mice (*n* = 8–9 mice/group). **f** Representative fluorescent micrographs of hippocampal ADtg-SB (left) and ADtg-MV (right) immunolabeled for GFAP^+^ astrocytes (red), 6E10^+^ human Aβ (white), FITC-dextran extravasation (green) and nuclei (blue). Increased extravasation of FITC-dextran is seen in vessels without 6E10^+^ Aβ aggregates (traced in yellow dotted lines) while less extravasation of FITC-dextran is seen in vessels with 6E10^+^ Aβ aggregates (traced in blue dotted lines). Individual channel micrographs are shown below. Data from individual mice in MV (orange dots) and SB (yellow dots) groups are shown, as well as *p* values (*p*_i_ = *p* value for interaction; *p*_MV_ = *p* value for MV intervention effect; *p*_g_ = *p* value for genotype effect). Fold increase and percentage decreases compared to control groups are shown in red. *p < 0.05, **p < 0.01, using two-way ANOVA with Holm-Sidak’s post hoc multiple comparisons correction, while the asterisk in parenthesis signifies an unpaired two-tailed Student *t* test. For Pearson’s *r* correlations, both *r* and *p* values, as well as 95% CI dashed lines are presented

Alterations in blood-brain barrier function are implicated in AD and were, thus, assessed by quantifying permeability of small (Texas Red-dextran 3 kD) and large (FITC-dextran 2000 kDa) molecules as evaluated by histology. Although there was a large variability in the sample data for FITC-dextran and Texas Red-dextran (Fig. 4a, b), a significant decrease in the hippocampal accumulation of Texas Red-dextran 3 kD and FITC-dextran 2000 kD were noted in mechanically ventilated ADtg mice and a non-significant increase in hippocampal accumulation of Texas Red-dextran 3 kD and FITC-dextran 2000 kD in the wild-type mice compared to the respective controls who did not undergo mechanical ventilation (Fig. 4a, b). There was a significant interaction between genotype and mechanical ventilation on the measured outcome of Texas Red-dextran 3 kD permeability (Fig. 4b). There was a significant positive correlation between percent of polymorphonuclear cells in the bronchoalveolar fluid and hippocampal accumulation FITC-dextran 2000 kD (Fig. 4d) and a significant negative correlation between cerebral soluble Aβ_1–42_ accumulation and fluorescence area of FITC-dextran 2000 kD (Fig. 4e). Representative microscopy with fluorescence staining is shown in Fig. 4c and f. Figure 4c is from one of the three animals who had markedly increased FITC-dextran 2000 kD accumulation following mechanical ventilation while Fig. 4f shows increased cerebral Aβ aggregates (white) in the non-mechanically ventilated ADtg mice associated with hippocampal GFAP+ reactive astrocytes (red) and localized FITC staining (green) in and near blood vessels. The mechanically ventilated mice exhibited reduced hippocampal permeability and 6E10-Aβ deposits near and inside blood vessels (Fig. 4f).

## Discussion

In this study, we found strong correlations between acute pulmonary inflammation after short-term mechanical ventilation and cerebral accumulation of soluble Aβ_1–40_, cerebral inflammation, and blood-brain barrier permeability. These data suggest that mechanical ventilation acutely promotes AD neuropathology by (i) increasing cerebral accumulation of the Aβ peptide and AD-associated neuroinflammation, notably TNF-α and IL-6 in ADtg mice with pre-existing AD pathology, and (ii) increasing AD-associated neuroinflammation, notably TNF-α and IL-6, and possibly blood-brain barrier permeability in wild-type mice without pre-existing AD pathology. These findings provide mechanistic insights into the well-documented clinical observations that associate mechanical ventilation with long-term cognitive decline and AD in particular [2, 17]. The non-significant trend towards increased blood-brain barrier permeability in the wild-type mice also suggests the presence of a common pathophysiology that contributes to both delirium, which is independently associated with accelerated cognitive decline, and AD [3, 8–10, 18, 19]. Further, the finding that soluble Aβ_1–40_, the most abundant Aβ isoform, increases with mechanical ventilation is noteworthy because prior studies have shown that soluble Aβ species significantly contribute to AD pathogenesis and even more so than insoluble Aβ aggregates [20–22].

Although causation is not yet proven through direct experimentation, the results of this investigation are consistent with prior studies that report a role for cerebral TNF-α in promoting cerebral Aβ accumulation [23, 24]; however, this effect is apparently independent of changes in plasma TNF-α, which did not change significantly following mechanical ventilation. This suggests that acute cerebral Aβ accumulation may be independent of systemic TNF-α concentrations and that mechanical ventilation or pulmonary inflammation may directly contribute to neuroinflammation, perhaps via local cerebral signaling mechanisms or by inducing alterations in Aβ clearance from the brain [25].

Our approach to assessing blood-brain barrier dysfunction involved in vivo administration of high (FITC-dextran 2000 kD) and low (Texas Red-dextran 3 kD) molecular weight tracers 30 min prior to sacrifice, followed by tissue extraction and histological analysis. Notably, the molecular weight of Aβ_1–42_ is approximately 4.4 kD and is thus closely approximated by the size of the Texas Red-dextran 3 kD tracer influx that was used in this study [26]. This approach allowed us to reveal the complex relationships between mechanical ventilation, cerebral Aβ accumulation, and blood-brain barrier permeability. Our results show a trend towards increased hippocampal blood-brain barrier permeability in the wild-type animals, which suggests that short-term mechanical ventilation may impair the function of neuroanatomical structures responsible for cognition in otherwise healthy brains. Since the blood-brain barrier plays a central role in maintaining normal cognition via clearance of Aβ and maintaining cerebral small vessel integrity, these findings suggest that mechanical ventilation may initiate neuropathology that leads to long-term cognitive impairment [8–10, 27–30]. Paradoxically, the ADtg mice showed significantly decreased blood-brain barrier permeability despite increased cerebral Aβ_1–40_ concentration. These unexpected results may suggest that Aβ exerts a protective effect that limits the influx of toxins from the vascular space into the brain parenchyma across the blood-brain barrier. Future studies are needed to assess whether leakage of tracer is more restricted in regions where Aβ are aggregated and to clarify whether this is related to altered expression of Aβ receptors or transporters within the blood-brain barrier.

This study has important limitations that should be considered. Although these data indicate statistical significance in three principal components of cognitive impairment—namely, accumulation of cerebral soluble Aβ_1–40_, increase in key cognition-relevant inflammatory biomarkers, and alteration of blood-brain barrier permeability—it is possible that future studies with larger sample sizes could reveal additional significant effects of mechanical ventilation on other Aβ isoforms that are less abundant than soluble Aβ_1–40._ Conversely, it is also possible that the trend towards increased blood-brain brain permeability in the wild-type mice reverses with larger sample sizes, notwithstanding the biological plausibility of increased blood-brain barrier permeability in systemic and systemic inflammatory conditions [7]. Additionally, as there were significant interactions between genotype and mechanical ventilation for cerebral IL-5, IL-10, and Texas Red-dextran 3 kD permeability, future studies should be designed to identify genotype-specific variables that may have influenced these outcomes.

Another potential limitation is the use of high dose of mechanical ventilation, i.e., 15 cc/kg tidal volumes, which may not reflect current clinical practice where lower tidal volume ventilation is generally considered the standard of care. However, these animals were also healthier and did not have any concurrent systemic infections or exposure to high doses of sedatives that could have increased the overall medical acuity. Subsequent research is needed to determine whether lower tidal volume ventilation decreases the burden of neurological injury or whether superimposed effects of systemic diseases such as sepsis alter the extent of neurological injury. Although the young mice used in this experiment do not necessarily reflect the demographic of older intensive care patients who undergo mechanical ventilation. The use of these animals with less baseline AD-pathology allowed us to clearly demonstrate the accelerated trajectory of AD-relevant neuropathology. Future studies are needed to study the effects of mechanical ventilation in older mice.

To simulate the real-life application of mechanical ventilation, the animals who underwent mechanical ventilation received sedation, while the non-mechanically ventilated animals did not receive sedation. The provision of sedation to the non-mechanically ventilated control group could alter respiratory drive and lead to additional confounding variables, such as hypoxemia or hypercapnia, which could independently alter cerebrovascular hemodynamics and the measured outcomes. We, thus, opted to avoid this effect of sedation in our control animals. The issue of sedation remains challenging and one that will need to be clarified in future investigations that strive to identify the independent effects of sedatives on the measured outcomes. Finally, the results of this study must be interpreted with caution as findings from pre-clinical studies are not invariably confirmed in clinical trials, and although significant efforts were made to minimize introduction of bias, including the use of blinded data analysts and concealed experimental group allocation, it may not be possible to fully eliminate the introduction of bias.

## Conclusions

In conclusion, our data indicate that short-term mechanical ventilation, a common critical care intervention, promotes neuropathology that is a characteristic of AD by increasing cerebral soluble Aβ_1–40_, promoting neuroinflammation, and altering blood-brain barrier permeability. Future studies are needed to further clarify specific molecular mechanisms that underlie these findings that will facilitate the development of neuroprotective mechanical ventilation strategies that could mitigate the risk of cognitive decline after critical illness.

## Additional files

Additional file 1: **Figure S1.** Mechanical ventilation-associated lung injury and cerebral Aβ concentrations in wild-type (WT) and transgenic Alzheimer’s Disease (ADtg) mice. **Figure S2.** Key cytokines in plasma of WT and ADtg following mechanical ventilation (MV). The Meso Scale Discovery (MSD) multiplex inflammatory assay performed on plasma and brain. **Figure S3.** IL-12p70 and IL-2. **Figure S4.** IFNγ and IL-4 cytokines. **Figure S5.** IL-15, IL-33, and IL-17A/F. **Figure S6.** MIP-1α, MCP-1 and MIP-2. **Figure S7.** KC/GRO and IP-10. **Figure S8.** Correlations between cerebral soluble Aβ and key brain cytokines in ADtg mice and correlations between soluble Aβ1–40 and Aβ1–42, and cerebral cytokines from age-matched ADtg mice subjected to MV or SB. **Figure S9.** Correlations between cerebral soluble Aβ and chemokines in SB and MV ADtg mice. Correlation between soluble Aβ1–40 and Aβ1–42 levels and the cerebral inflammatory cytokines performed from age-matched ADtg mice. **Figure S10.** Correlations between cerebral insoluble Aβ and cerebral inflammatory biomarkers in SB and MV ADtg mice. Correlation between insoluble Aβ1–40 and Aβ1–42 and inflammatory cytokines from brains of ADtg mice subjected to MV or SB. **Figure S11.** Hippocampal blood-brain barrier permeability of WT and ADtg mice following MV. A. Representative confocal images of Texas Red-dextran tracer in WT (top) and ADtg (bottom) mice under SB condition. B. Correlation between Texas Red-dextran hippocampal leakage and PMNs in the bronchoalveolar lavage fluid. C-D. Correlation between soluble Aβ1–40 in age-matched ADtg mice, and C. FITC-dextran hippocampal leakage, and D. Texas Red-dextran hippocampal leakage. E. Correlation of soluble Aβ1–42 in age-matched ADtg mice and Texas Red-dextran hippocampal permeability. F-G. Correlation between insoluble Aβ1–40 in ADtg mice, and F. FITC-dextran hippocampal leakage, and G. Texas Red-dextran hippocampal leakage. H-I. Correlation between insoluble Aβ1–42 and: H. FITC-dextran hippocampal leakage, and I. Texas Red-dextran hippocampal permeability. (PDF 8663 kb)

Additional file 2: **Table S1.** Statistical analysis of mechanical ventilation data. O2 = oxygen saturation; PMNs = polymorphonuclear cells; dF = degrees of freedom; *F* = *F* value. Two-way ANOVA statistical analysis for mechanical ventilation data of all four experimental groups. **Table S2.** MSD analysis of brain cytokines in wild-type and ADtg mice. MSD = Meso Scale Discovery (MSD) multiplex inflammatory assay; MV = mechanical ventilation; SB = spontaneous breathing. Average cytokine levels are shown per group, per genotype. The difference between groups is shown as % change and fold change (FC). For each cytokine, two-way ANOVA was applied and *p*-values are also shown for ad-hoc Sidak’s posttest analysis. Unpaired *t* test was applied showing no statistical difference between the genotypes in cytokine levels change (% and fold) with MV (*p* = 0.579). **Table S3.** MSD analysis of brain chemokines in wild-type and ADtg mice. MSD = Meso Scale Discovery (MSD) multiplex inflammatory assay; MV = mechanical ventilation; SB = spontaneous breathing. Average cytokine levels are shown per group, per genotype. The difference between groups is shown as % change and fold change (FC). For each chemokine, two-way ANOVA was applied and p-values are also shown for ad-hoc Sidak’s posttest analysis. Unpaired t-test was applied showing no statistical difference between the genotypes in cytokine levels change (% and fold) with MV (*p* = 0.129). **Table S4.** Statistical analysis of BBB permeability data. BBB = blood-brain barrier; FITC % = FITC-dextran % area; Texas Red % = Texas Red-dextran % area; dF = degrees of freedom; *F* = *F* value. Two-way ANOVA statistical analysis for BBB Permeability data of all four experimental groups. (DOCX 162 kb)

## Abbreviations

AD: Alzheimer’s disease
ADtg: Double transgenic APP_SWE_/PSEN1_∆E9_
Aβ: Amyloid β
MSD: Meso Scale Discovery
