# Supplementary figures and images for: Acute neuropathological consequences of short-term mechanical ventilation in wild-type and Alzheimer’s disease mice
Source: Crit Care. 2019 Feb 22;23:63. doi: 10.1186/s13054-019-2356-2 (PMC6387486; doi:10.1186/s13054-019-2356-2)

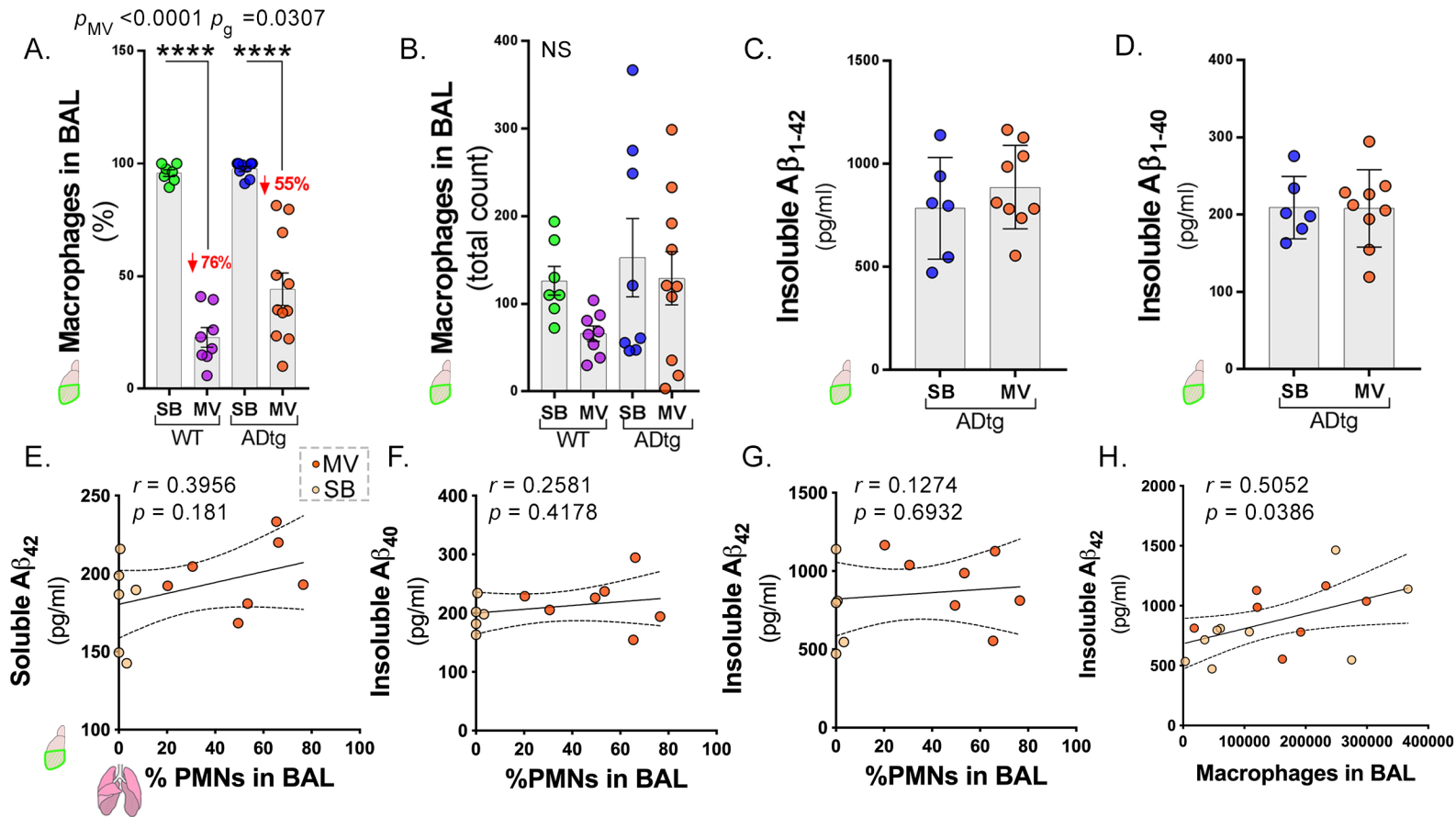

Suppl. Fig.1

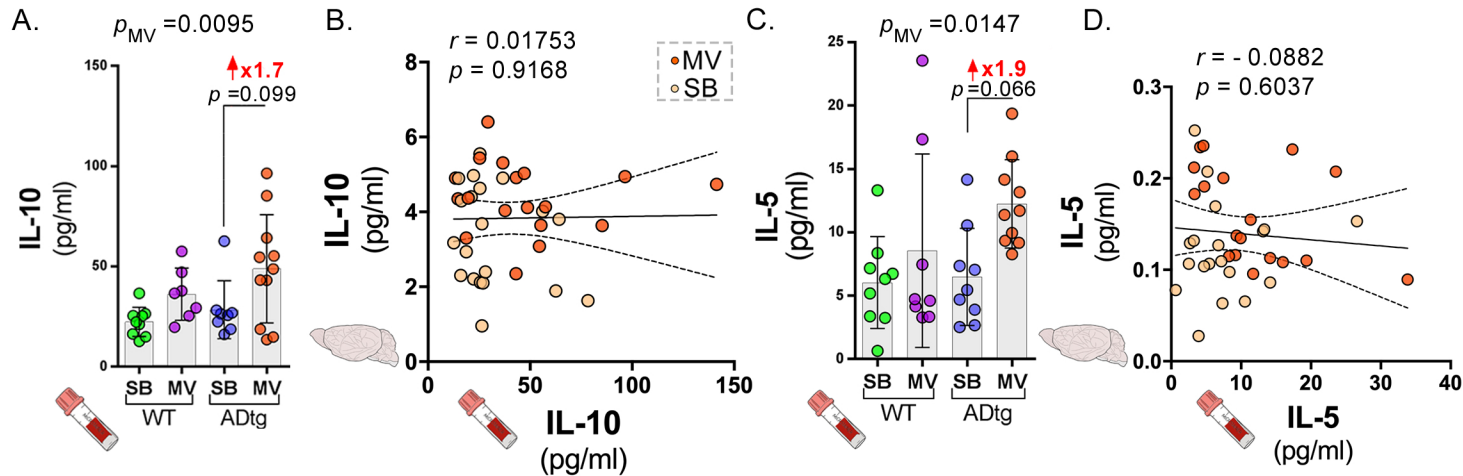

Suppl. Fig 2

A.  $p_{MV} = 0.0013$   $p_g < 0.0001$

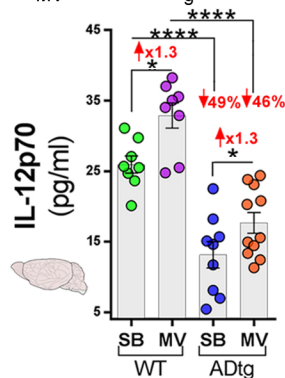

$p_i = 0.0214$   $p_{MV} = 0.0288$

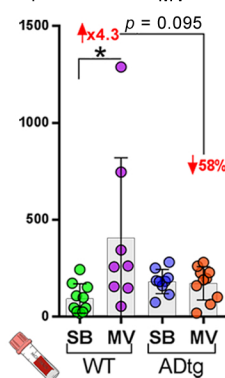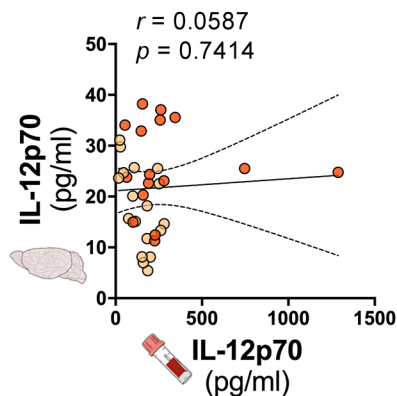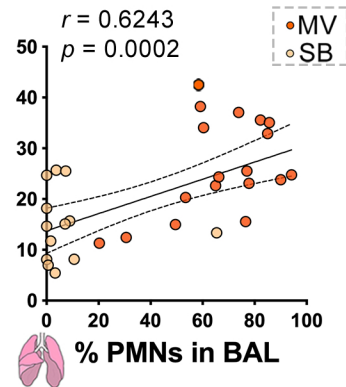

B.

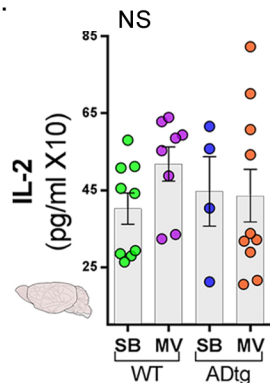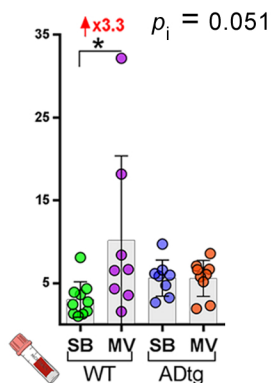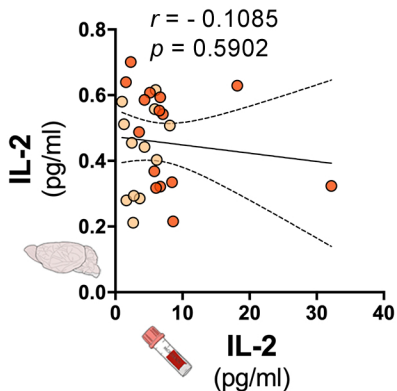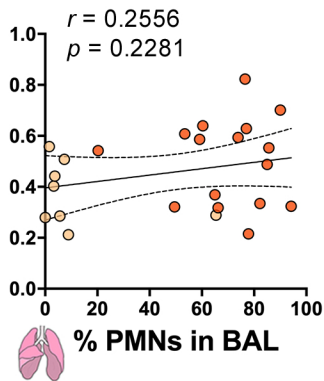

Suppl. Fig. 3

A.

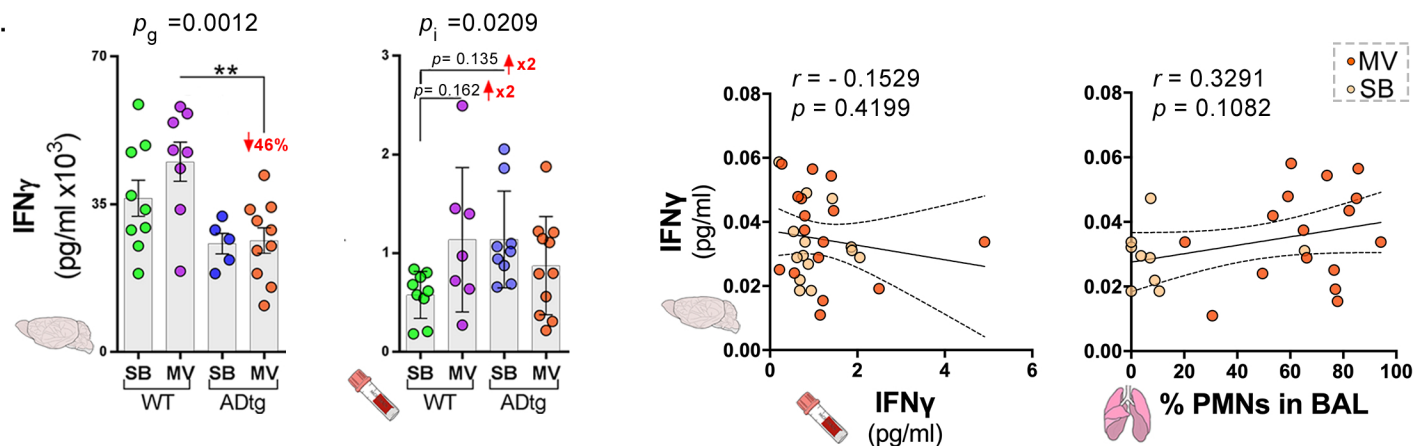

B.

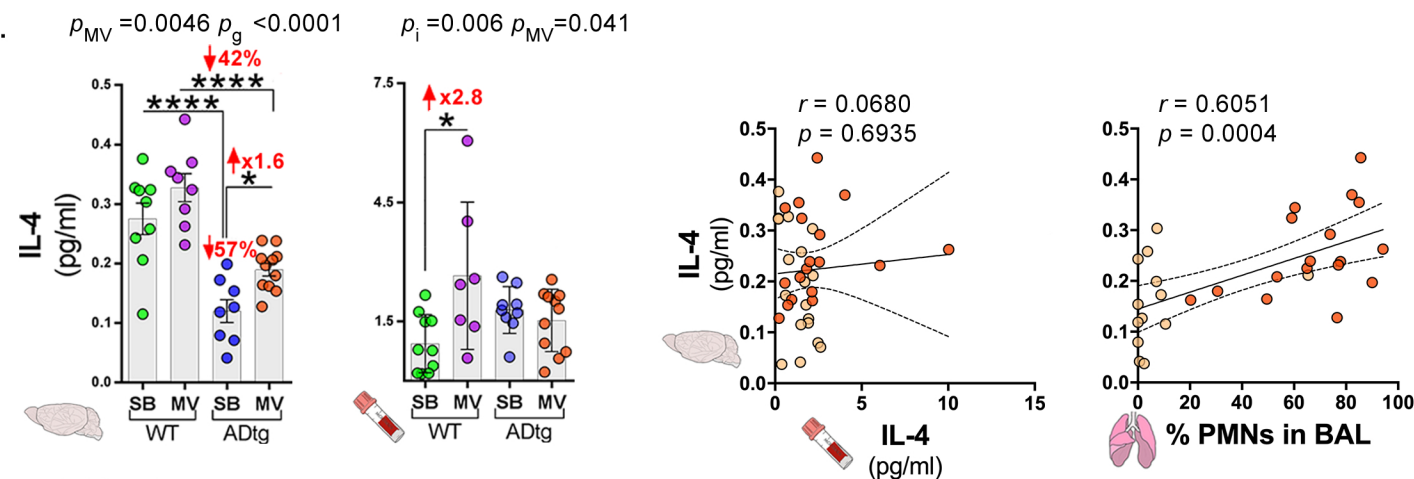

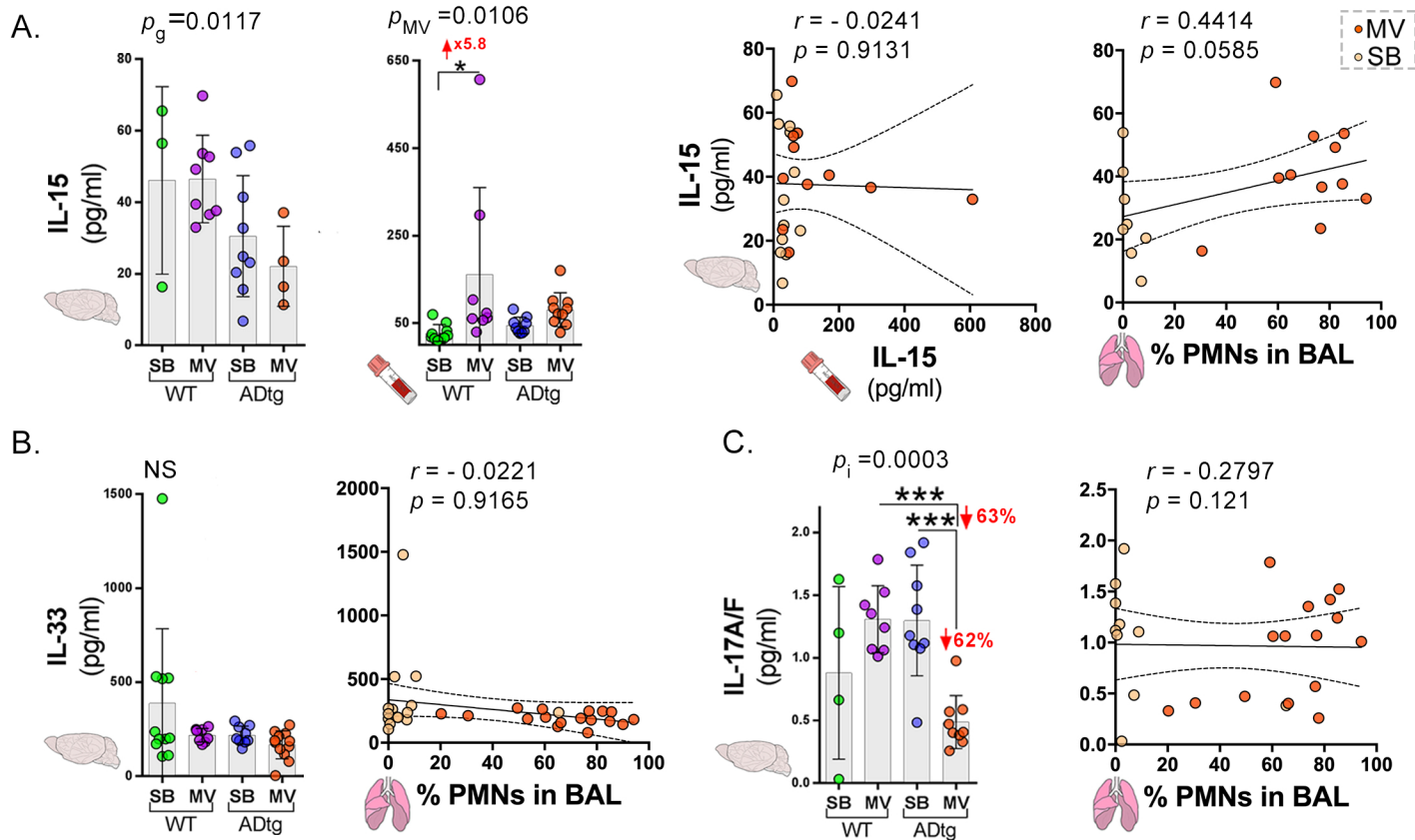

Suppl. Fig. 5

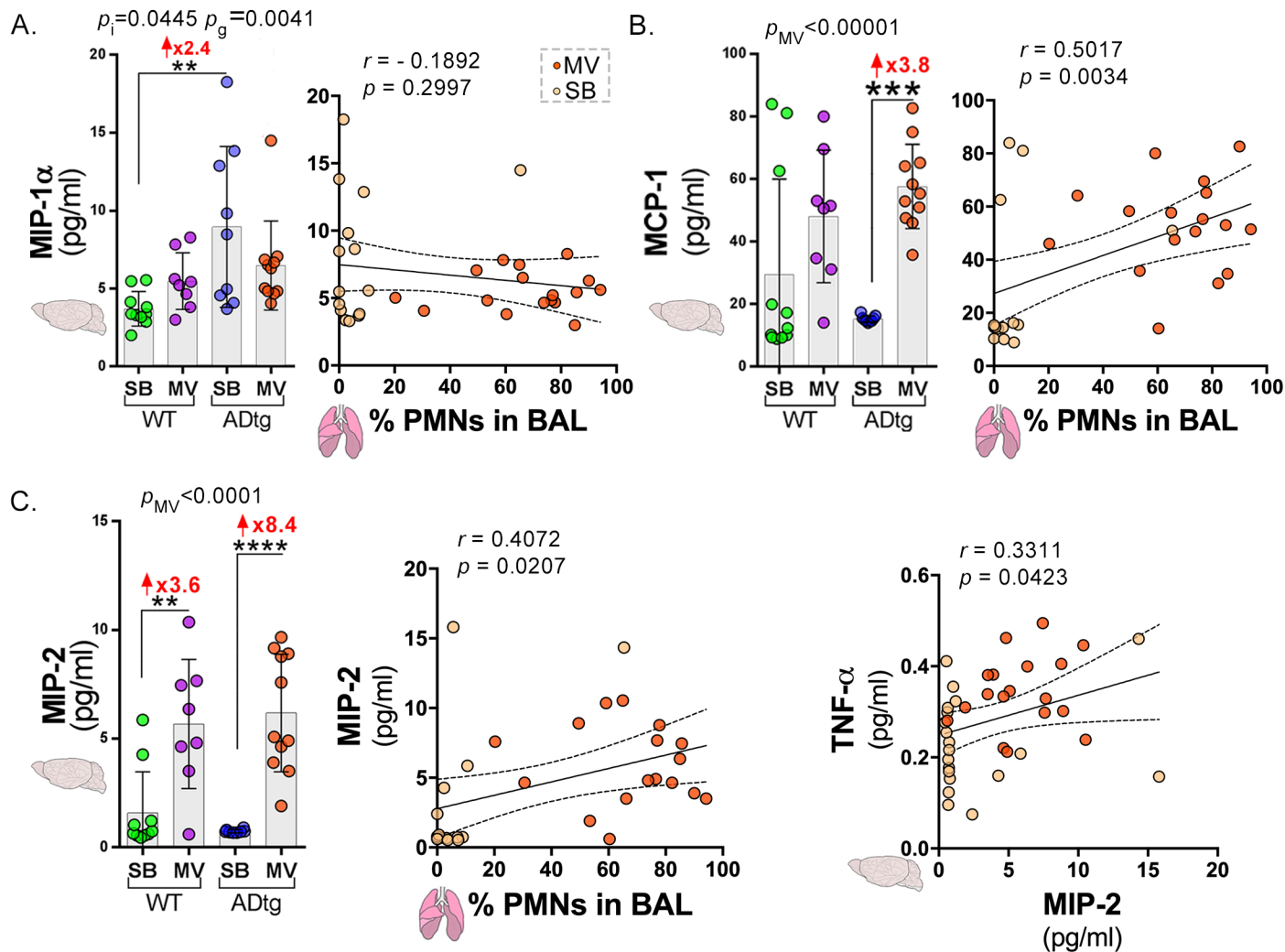

Suppl. Fig. 6

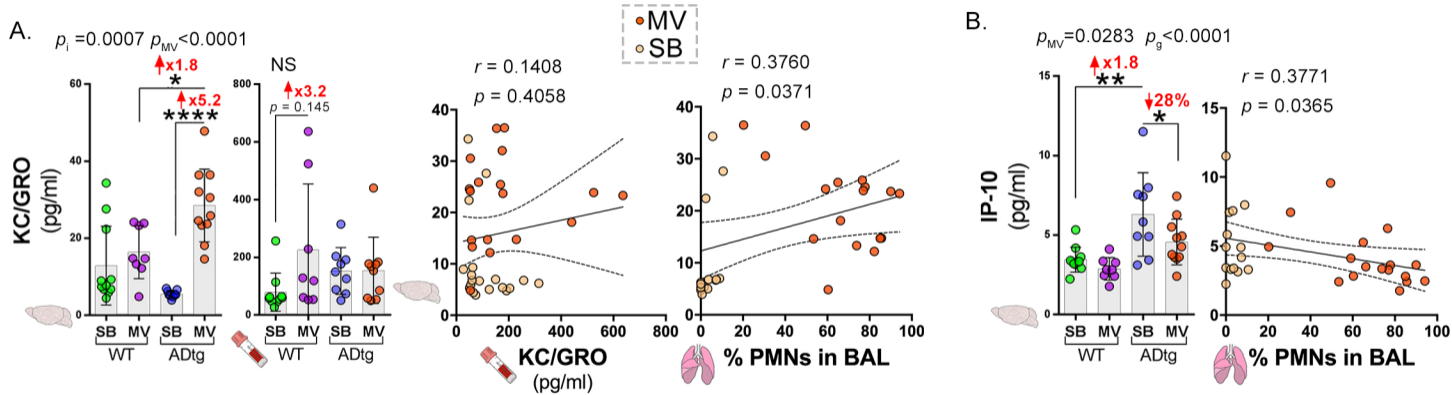

Suppl. Fig. 7

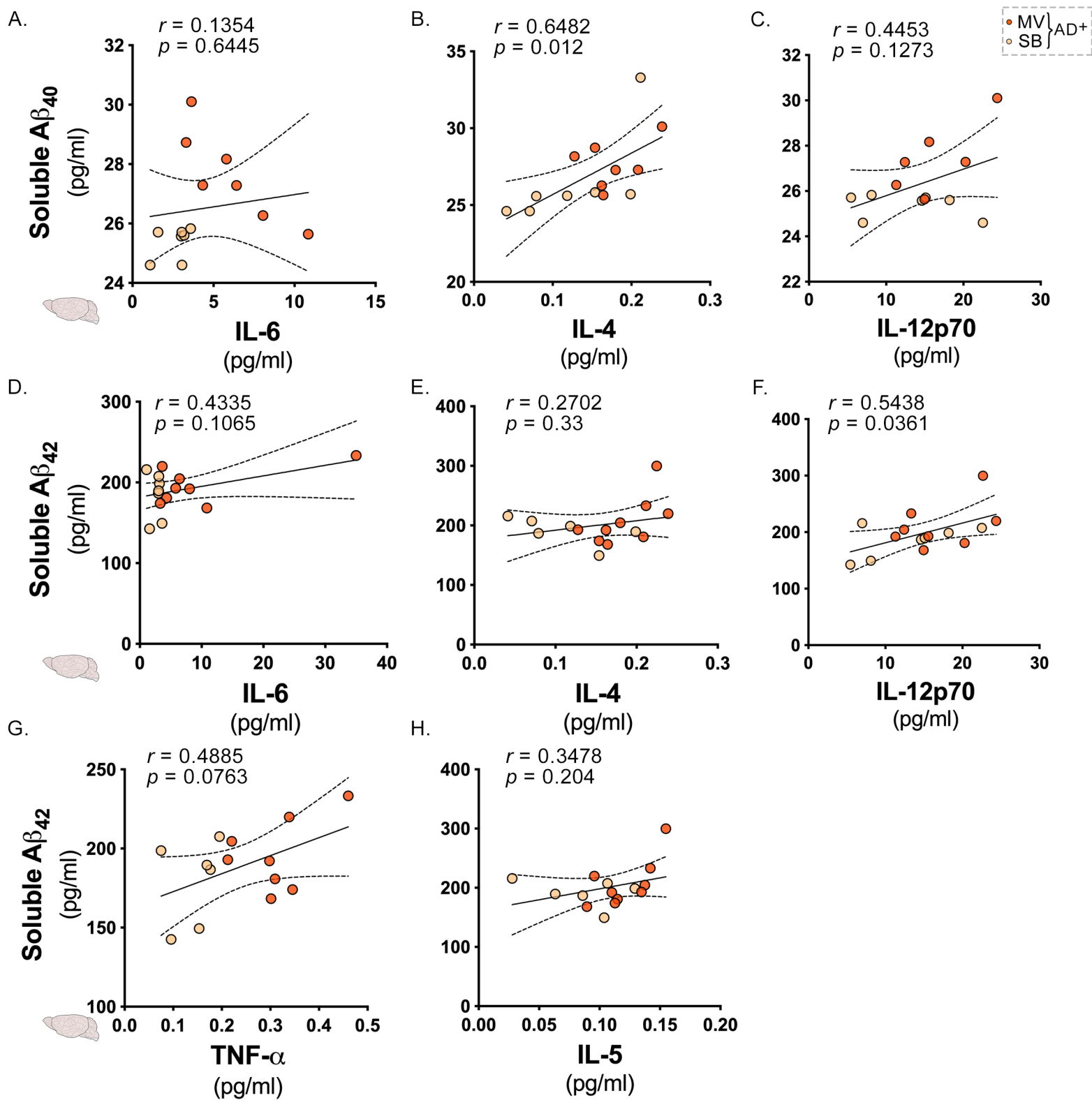

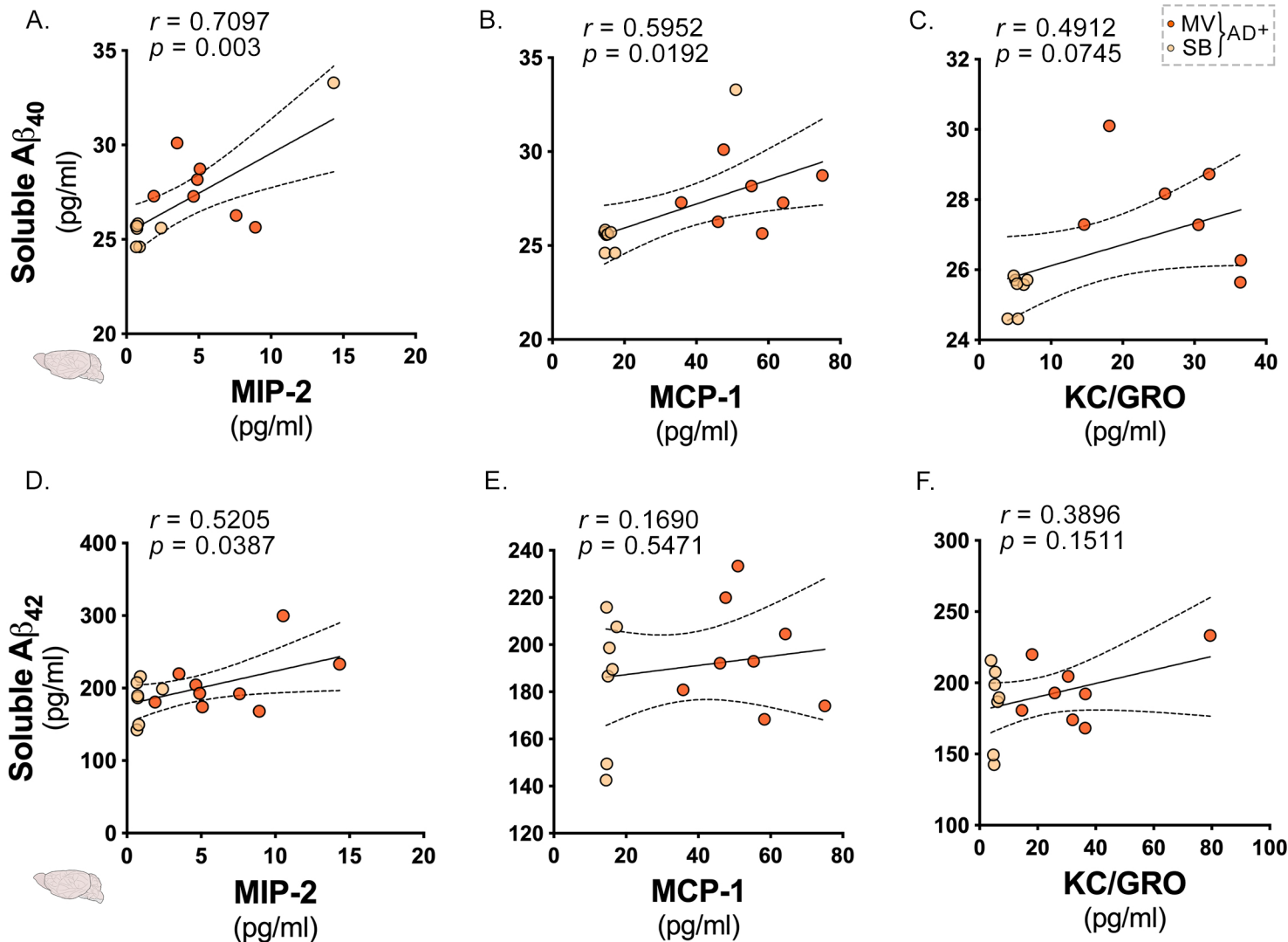

Suppl. Fig. 9

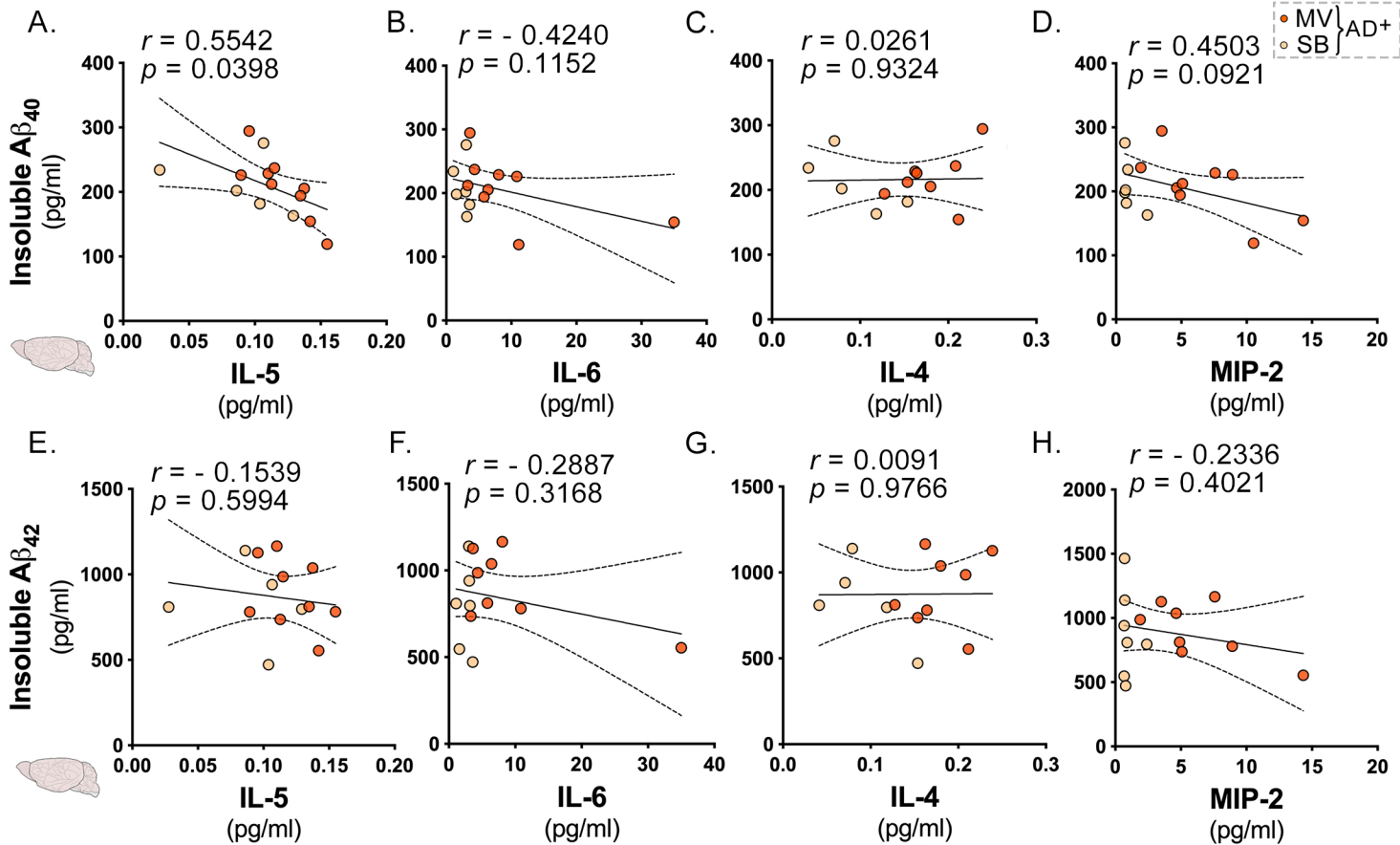

Suppl. Fig. 10

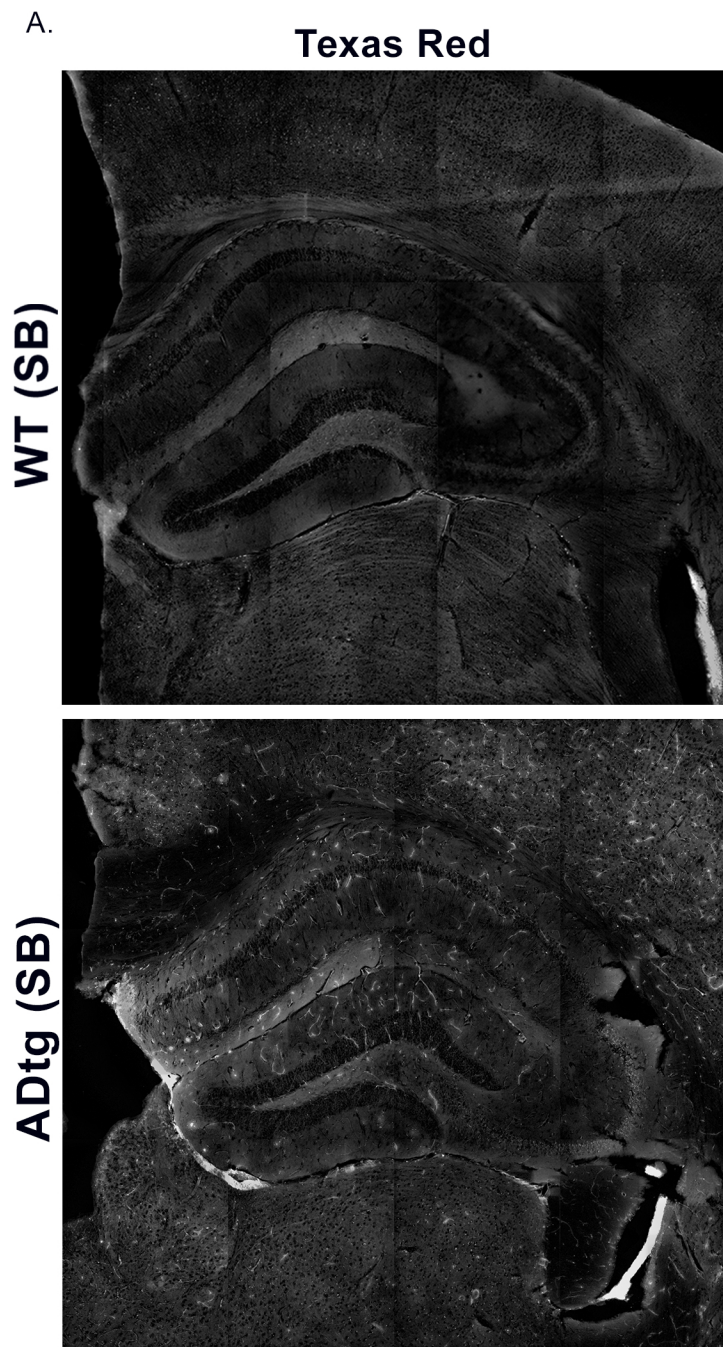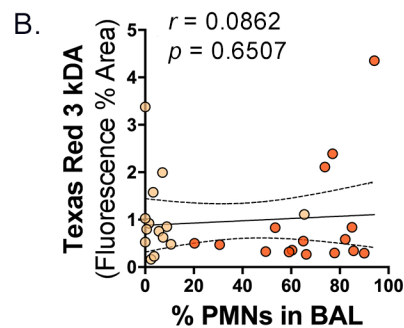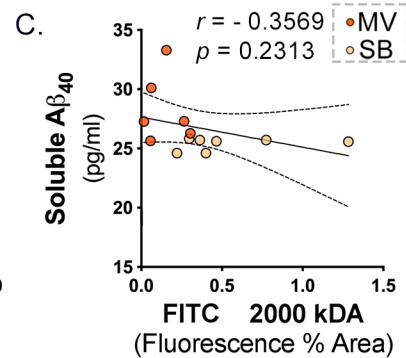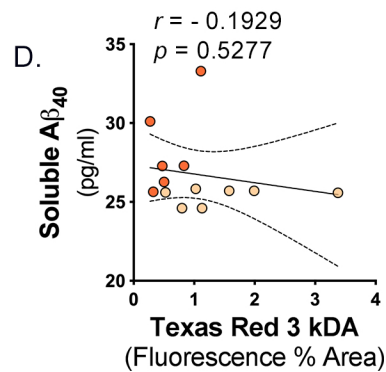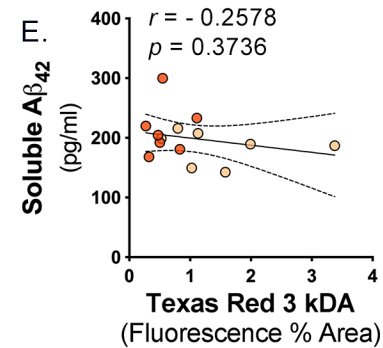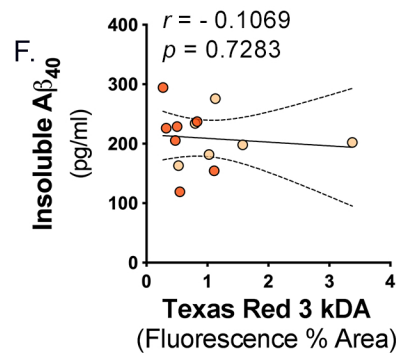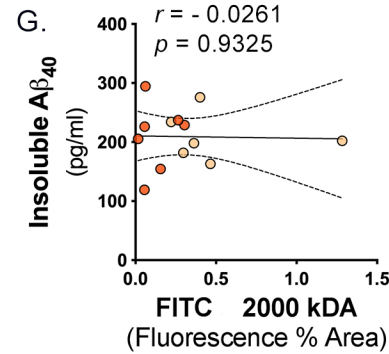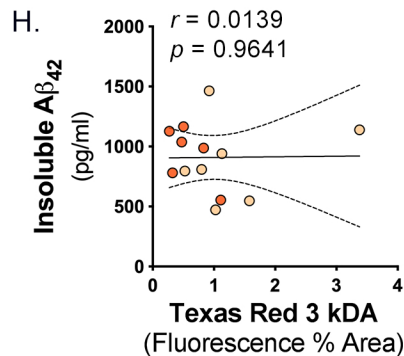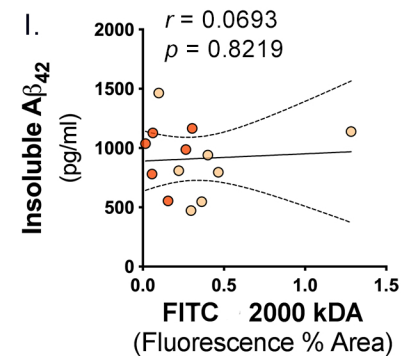

Supplement: Supplementary file 1 — Figure S1. Mechanical ventilation-associated lung injury and cerebral Aβ concentrations in wild-type (WT) and transgenic Alzheimer’s Disease (ADtg) mice. Figure S2. Key cytokines in plasma of WT and ADtg following mechanical ventilation (MV). The Meso Scale Discovery (MSD) multiplex inflammatory assay performed on plasma and brain. Figure S3. IL-12p70 and IL-2. Figure S4. IFNγ and IL-4 cytokines. Figure S5. IL-15, IL-33, and IL-17A/F. Figure S6. MIP-1α, MCP-1 and MIP-2. Figure S7. KC/GRO and IP-10. Figure S8. Correlations between cerebral soluble Aβ and key brain cytokines in ADtg mice and correlations between soluble Aβ1–40 and Aβ1–42, and cerebral cytokines from age-matched ADtg mice subjected to MV or SB. Figure S9. Correlations between cerebral soluble Aβ and chemokines in SB and MV ADtg mice. Correlation between soluble Aβ1–40 and Aβ1–42 levels and the cerebral inflammatory cytokines performed from age-matched ADtg mice. Figure S10. Correlations between cerebral insoluble Aβ and cerebral inflammatory biomarkers in SB and MV ADtg mice. Correlation between insoluble Aβ1–40 and Aβ1–42 and inflammatory cytokines from brains of ADtg mice subjected to MV or SB. Figure S11. Hippocampal blood-brain barrier permeability of WT and ADtg mice following MV. A. Representative confocal images of Texas Red-dextran tracer in WT (top) and ADtg (bottom) mice under SB condition. B. Correlation between Texas Red-dextran hippocampal leakage and PMNs in the bronchoalveolar lavage fluid. C-D. Correlation between soluble Aβ1–40 in age-matched ADtg mice, and C. FITC-dextran hippocampal leakage, and D. Texas Red-dextran hippocampal leakage. E. Correlation of soluble Aβ1–42 in age-matched ADtg mice and Texas Red-dextran hippocampal permeability. F-G. Correlation between insoluble Aβ1–40 in ADtg mice, and F. FITC-dextran hippocampal leakage, and G. Texas Red-dextran hippocampal leakage. H-I. Correlation between insoluble Aβ1–42 and: H. FITC-dextran hippocampal leakage, and I. Texas Red-dex [file 13054_2019_2356_MOESM1_ESM.pdf]
