# Supplementary material for: Acute neuropathological consequences of short-term mechanical ventilation in wild-type and Alzheimer’s disease mice
Source: Crit Care. 2019 Feb 22;23:63. doi: 10.1186/s13054-019-2356-2 (PMC6387486; doi:10.1186/s13054-019-2356-2)
Supplement: Supplementary file 2 — Table S1. Statistical analysis of mechanical ventilation data. O2 = oxygen saturation; PMNs = polymorphonuclear cells; dF = degrees of freedom; F = F value. Two-way ANOVA statistical analysis for mechanical ventilation data of all four experimental groups. Table S2. MSD analysis of brain cytokines in wild-type and ADtg mice. MSD = Meso Scale Discovery (MSD) multiplex inflammatory assay; MV = mechanical ventilation; SB = spontaneous breathing. Average cytokine levels are shown per group, per genotype. The difference between groups is shown as % change and fold change (FC). For each cytokine, two-way ANOVA was applied and p-values are also shown for ad-hoc Sidak’s posttest analysis. Unpaired t test was applied showing no statistical difference between the genotypes in cytokine levels change (% and fold) with MV (p = 0.579). Table S3. MSD analysis of brain chemokines in wild-type and ADtg mice. MSD = Meso Scale Discovery (MSD) multiplex inflammatory assay; MV = mechanical ventilation; SB = spontaneous breathing. Average cytokine levels are shown per group, per genotype. The difference between groups is shown as % change and fold change (FC). For each chemokine, two-way ANOVA was applied and p-values are also shown for ad-hoc Sidak’s posttest analysis. Unpaired t-test was applied showing no statistical difference between the genotypes in cytokine levels change (% and fold) with MV (p = 0.129). Table S4. Statistical analysis of BBB permeability data. BBB = blood-brain barrier; FITC % = FITC-dextran % area; Texas Red % = Texas Red-dextran % area; dF = degrees of freedom; F = F value. Two-way ANOVA statistical analysis for BBB Permeability data of all four experimental groups. (DOCX 162 kb) [file 13054_2019_2356_MOESM2_ESM.docx]

**Suppl. Table 1. Statistical Analysis of Mechanical Ventilation Data.**

| **Data** | **two-way ANOVA** | | | |
| --- | --- | --- | --- | --- |
|  | ***p*-value** | | **dF** | **F** |
| O2 | Interaction | 0.0783 | (4, 75) | 2.189 |
|  | Time | <0.0001 |  | 7.149 |
|  | Genotype | 0.0003 |  | 14.31 |
| %PMNs | Interaction | 0.1035 | (1,29) | 2.826 |
|  | MV | <0.0001 |  | 182.8 |
|  | Genotype | 0.0547 |  | 4.009 |
| % Macrophages | Interaction | 0.066 | (1, 31) | 3.631 |
|  | MV | <0.0001 |  | 151.7 |
|  | Genotype | 0.0307 |  | 5.126 |
| # Macrophages | Interaction | 0.5408 | (1, 29) | 0.3831 |
|  | MV | 0.1693 |  | 1.986 |
|  | Genotype | 0.1428 |  | 2.269 |

**Suppl. Table 2. MSD Analysis of Brain Cytokines in WT and ADtg mice.**


**Suppl. Table 3. MSD Analysis of Brain Chemokines in Wild-Type and ADtg mice.**

**Suppl. Table 4. Statistical Analysis of BBB Permeability Data.**

| **Data** | **two-way ANOVA** | | | |
| --- | --- | --- | --- | --- |
|  | ***p*-value** | | **dF** | **F** |
| FITC% (2000 kDa) | Interaction | 0.066 | (1, 28) | 3.659 |
|  | MV | 0.2302 |  | 1.505 |
|  | Genotype | 0.1644 |  | 2.039 |
| Texas Red % (3 kDa) | Interaction | 0.0039 | (1, 27) | 9.961 |
|  | MV | 0.6327 |  | 0.2336 |
|  | Genotype | 0.8968 |  | 0.01713 |
